# Associations between Dietary Antioxidant Intake and Metabolic Syndrome

**DOI:** 10.1371/journal.pone.0130876

**Published:** 2015-06-22

**Authors:** Jie Wei, Chao Zeng, Qian-yi Gong, Xiao-xiao Li, Guang-hua Lei, Tu-bao Yang

**Affiliations:** 1 Department of Epidemiology and Health Statistics, School of Public Health, Central South University, Changsha, Hunan Province, China, 410008; 2 Department of Orthopaedics, Xiangya Hospital, Central South University, Changsha, Hunan Province, China, 410008; RWTH Aachen, GERMANY

## Abstract

**Background:**

The objective of this study was to evaluate the association between dietary antioxidant intake (carotenoid, vitamin C, E and selenium) intake and metabolic syndrome (MS).

**Method:**

This cross-sectional study included 2069 subjects undergoing a regular health checkup. Biochemical test results and data on dietary intakes were collected for analysis. Adjustment for energy intake and multi-variable logistic regression were performed to determine adjusted odds ratios (ORs) and corresponding 95% confidence intervals (95%CI) for the relationship between dietary antioxidants intake and MS. The lowest quartile of antioxidant intake was regarded as the reference category.

**Result:**

Dietary vitamin C intake (P values for trend were 0.02 in energy adjusted analysis and 0.08 in multivariable adjusted analysis) had a negative association with MS, as did selenium intake in the second quartile (energy adjusted OR: 0.60, 95%CI: 0.43 to 0.85; multivariable adjusted OR: 0.60, 95%CI: 0.43 to 0.86). However, there was no significant relationship between dietary carotenoid and vitamin E intake and MS.

**Conclusion:**

Subjects with low intake of vitamin C might be predisposed to development of MS, while dietary selenium intake had a moderate negative association with MS. Dietary carotenoid and vitamin E intake was not associated with MS.

## Introduction

Metabolic syndrome (MS) is a pathological state of energy utilization and storage, and is characterized by the following clinical features: abdominal (central) obesity, elevated blood pressure (BP), hyperglycemia, high serum triglyceride levels and low level of high density lipoprotein (HDL). MS is commonly considered to be a risk factor for cardiovascular disease, diabetes mellitus and death. [1] In recent years, the prevalence of MS has increased significantly both in developed and developing countries, to approximately 25% in the United States, [2] and around 13.2% to 15.8% in China. [3–6] Thus, MS has become a major global public health issue and had attracted increasing attention from clinical physicians and medical science researchers.

Previous studies have reported the impact of oxidative stress and inflammation on several chronic diseases, especially on MS, cardiovascular disease, and diabetes mellitus. [7–10] A limited number of studies [11–14] have explored the relationship between dietary antioxidants intake and MS, but the conclusions have been inconsistent. Puchau et al. [8] suggested that total dietary antioxidant capacity may be a potential early indicator in those at risk of developing MS. Bian et al. [11], Li et al. [12] and Motamed et al. [13] all denied the existence of a significant relationship between dietary antioxidants intake and MS. However, the studies of Bian et al. [11] and Li et al. [12] had small sample size (258 and 550 subjects, respectively), while the results from Li et al. were only adjusted for age and sex. Ford et al. [14] reported a significant difference in dietary vitamin A intake between MS and non-MS subjects, but there was no difference in vitamin C and E intake between the two groups. Additionally, these results were not adjusted for other confounding factors, such as the basic characteristics of the included subjects and energy intake.

The objective of this study was to evaluate the association between dietary antioxidants intake (vitamin C, vitamin E, carotenoids and selenium) and MS with adjustment for potential confounders. This cross-sectional study was based on data from a large sample size of Chinese adults.

## Methods

### Subjects

The Xiangya Hospital Health Management Center is the one of the largest health management centers in Hunan Province, China. Study participants were recruited when they attended the Center for regular health examination between October 2013 and January 2014. Subjects aged over 18 years who voluntarily underwent a blood biochemistry test anthropometric measurement (e.g., height, weight, waist circumference), and the dietary assessment with a structured questionnaire, covering demographic information and data on lifestyle habits (cigarette smoking, alcohol drinking, medication use, nutritional supplementation and regular exercise) were qualified for this study. A total of 2440 subjects had a general blood biochemical test. Individuals with missing data on anthropometric parameters (n = 9) or dietary intake assessment (n = 362) were excluded from the study, and 2069 subjects were ultimately included in the present research.

Written informed consent was obtained from all subjects in this study. The protocol of this study was approved by the Ethics Committees on Research of the Xiangya Hospital, Central South University.

### Dietary assessment

A specially designed semi-quantitative food frequency questionnaire (SFFQ) was used to evaluate dietary intake. This included 63 food items that are popular and commonly consumed in Hunan Province, China. For each food item, participants were asked how frequently they consumed the food (never, once a month, two to three times a month, one to three times a week, four to five times a week, once a day, twice a day, or more than twice a day) and how much on average they consumed for each time in the past year (less than 100g, 100–200g, 201–300g, 301–400g, 401g–500g, and more than 500g). Color pictures of food samples with labeled weights were provided to participants to help them make choices more easily and accurately. The response rate for the SFFQ was 86.2% in the present study. The validity of the SFFQ was estimated by comparing the results with the 24-hour dietary recall method for the similar population. A total of 91 subjects’ dietary intake were assessed by both the SFFQ and 24-hour dietary recall method in a face-to-face review at the same session. Pearson correlation coefficients (unadjusted) between the SFFQ and the 24-h recalls data for selenium, carotenoid vitamin, C, and vitamin E intake were 0.35, 0.14, 0.27 and 0.33, respectively. The correlation coefficients with adjustment for total energy intake were also computed by the residual model. [15] After adjusted for energy, the correlation coefficients decreased a little (r = 0.21 for selenium, r = 0.14 for carotenoid, r = 0.20 for vitamin C and r = 0.16 for vitamin E). The Chinese Food Composition Table [16] was used to calculate the individual composition of macronutrients and micronutrients of the included food items.

### Metabolic Syndrome

According to the current definition of MS in the American Heart Association Guidelines, [17] subjects with the presence of any three of the following risk factors could be considered as suffering from MS: waist circumference ≥ 102 cm in men and ≥ 88 cm in women; triglycerides ≥ 150 mg/dL (1.7 mmol/L); HDL cholesterol < 40 mg/dL (1.03 mmol/L) in men and < 50 mg/dL (1.29 mmol/L) in women; systolic BP (SBP)≥ 130 mm Hg, diastolic BP (DPB) ≥85 mm Hg, or currently using antihypertensive medication; and fasting glucose ≥ 100 mg/dL (5.6 mmol/L) or currently undergoing drug treatment for blood glucose control.

Waist circumference was measured with a tape measure at the level of the umbilicus. SBP and DBP were measured using an electronic sphygmomanometer. All blood samples were drawn after a 12-hour overnight fast and were kept at 4°C until analysis. Serum fasting glucose was measured using the hexokinase (HK) measurement method, while total triglycerides was determined by the glucose oxidase-peroxidase (GPO-POD) method, and HDL cholesterol was assessed by direct assay. All blood analyses were performed on a Beckman Coulter AU 5800 (Beckman Coulter Inc., Brea, CA, USA).

### Statistical analysis

Continuous data are expressed as mean ± standard deviation, and category data as a percentage. Differences in continuous data were evaluated by the student *t*-test (normally distributed data) or the Mann-Whitney *U* test (non-normally distributed data). Differences in the qualitative data were assessed by the chi-squared test. The odds ratios (ORs) with 95% confidence intervals (CIs) for the association between dietary antioxidants intake (Vitamin C, E, A, retinol, carotenoid and selenium) and MS were calculated for each quartile of antioxidants intake, with the lowest quartile regarded as the reference category. Energy intake was used for calculation of the adjusted OR for each quartile of antioxidants intake. In addition, a multivariable logistic regression analysis, including age, sex, cigarette smoking, alcohol drinking, nutritional supplement, activity level, dietary energy intake, fiber intake and protein intake, was used to assess the associations between antioxidants intake and MS.

The association between dietary antioxidants intake and clinical features of MS were also examined using logistic regression analysis with adjustment as before.

Tests for linear trends were conducted using logistic regression with a median variable of antioxidants level in each category. All data analyses were performed using SPSS 17.0 (SPSS Inc., Chicago, IL, USA), and P≤0.05 was considered to indicate statistical significance. All tests were two tailed.

## Result

A total of 2069 subjects (1109 males and 960 females) aged from 18 to 84 years were included in this cross-sectional study. In accordance with the American Heart Association Guidelines, there were 351 subjects (17.0%) who were diagnosed with MS. The mean daily dietary intake (not including supplementation) of carotenoid, vitamin C, vitamin E and selenium were 4548.97 μg, 108.01 mg, 29.99 mg and 47.22 μg, respectively, which was approximately equal to or slightly higher than the recommended nutrient intake (RNI) in China. [18] The basic characteristics of subjects with or without MS are listed in Tables 1 and 2. A comparison of the two groups showed significant difference in age, body mass index, waist circumference, BP, serum triglycerides, HDL, and blood glucose. There were no significant differences regarding sex, cigarette smoking, alcohol drinking, activity level and nutritional supplementation between subjects with MS and without MS.

**Table 1:** Basic characteristics of the study population (n = 2069).

Basic characteristics	MS	Non-MS	P
N (%)	351 (17)	1718 (83)	-
Age (years)	52.39±9.09	50.69±9.53	0.01
Sex (%)			0.10
Male	57.5	52.8	
Female	42.5	47.2	
Cigarette smoking (%)			0.27
Yes	27.6	24.8	
No	72.4	75.2	
Alcohol drinking (%)			0.20
Yes	39.0	35.4	
No	61.0	64.6	0.91
Activity level (h/week)	2.59±3.76	2.64±3.81	
Nutritional supplementary (%)			0.17
Yes	27.9	31.6	
No	72.1	68.4	
BMI (kg/m2)	26.91±3.12	23.95±3.00	0.00
Waist circumference (cm)	90.76±7.79	82.53±8.37	0.00
Systolic blood pressure (mmHg)	137.46±16.22	123.61±16.43	0.00
Diastolic blood pressure (mmHg)	87.90±10.88	78.12±11.38	0.00
Triglyceride (mmol/L)	3.13±2.27	1.57±1.39	0.00
HDL-C (mmol/L)	1.24±0.29	1.54±0.37	0.00
Fasting serum glucose (mmol/L)	6.62±1.92	5.46±1.32	0.00

MS: metabolic syndrome, BMI: body mass index, HDL: high density lipoprotein.

**Table 2:** Dietary intake characteristics of study population.

	MS (n = 351)	Non-MS (n = 1718)	P
Dietary energy intake (Kcal/day)	1824.85±992.74	1739.59±849.39	0.29
Dietary fiber intake (g/day)	19.28±16.23	19.02±15.87	0.92
Dietary protein intake (/day)	74.49±47.68	71.25±43.07	0.43
Dietary carotenoid intake (μg/day)	4743.74±4771.63	4509.17±3890.52	0.77
Dietary vitamin C intake (mg/day)	104.16±79.14	108.80±84.43	0.16
Dietary vitamin E intake (mg/day)	30.67±18.28	29.84±15.36	0.53
Dietary selenium intake (μg/day)	48.54±29.77	46.95±27.63	0.68

MS: metabolic syndrome.

The energy intake-adjusted associations and multivariable adjusted associations between dietary antioxidants intake and MS are displayed in Table 3. There was no association between dietary carotenoid intake and MS according to the energy adjusted ORs and the multivariable adjusted ORs (P for trend = 0.59 and 0.77, respectively). Both the energy adjusted ORs and the multivariable adjusted ORs indicated that dietary vitamin C intake was inversely associated with MS in the second quartile (energy adjusted OR: 0.64, 95%CI: 0.46 to 0.88, P = 0.01; multivariable adjusted OR: 0.60, 95%CI: 0.42 to 0.85, P = 0.01) and in the highest quartile of intake (energy adjusted OR: 0.65, 95%CI: 0.47 to 0.90, P = 0.01; multivariable adjusted OR: 0.64, 95%CI: 0.43 to 0.94, P = 0.02) compared with the lowest quartile (P values for trend: 0.02 and 0.08, respectively). Dietary selenium intake in second quartile also had a negative association with MS (energy adjusted OR: 0.60, 95%CI: 0.43 to 0.85, P = 0.00; multivariable adjusted OR: 0.60, 95%CI: 0.43 to 0.86, P = 0.01) compared with the reference. However, neither energy adjusted ORs nor multivariable adjusted ORs suggested a significant relationship between dietary vitamin E intake and MS.

**Table 3:** Multivariable-adjusted relationship between dietary antioxidants (carotenoid, vitamin C, vitamin E and selenium) intake and MS.

	Quartiles of antioxidants				P for trend
	Q1 (lowest)	Q2	Q3	Q4 (highest)	-
Median carotenoid intake (μg/day)	1159.17	2440.02	4247.66	10053.73	-
MS (%)	18.2	14.5	18.0	17.2	-
Energy adjusted OR (95%CI)	1.00 (reference)	0.72(0.52, 1.01)	0.93 (0.67, 1.28)	0.81 (0.57, 1.15)	0.59
P value	-	0.06	0.64	0.24	-
Multivariable-adjusted OR (95%CI)	1.00 (reference)	0.73 (0.52, 1.03)	0.93 (0.66, 1.31)	0.83 (0.53, 1.29)	0.77
P value	-	0.07	0.66	0.41	-
Median vitamin C intake (mg/day)	41.97	70.65	108.41	182.07	-
MS (%)	20.3	14.3	17.6	15.7	-
Energy adjusted OR (95%CI)	1.00 (reference)	0.64 (0.46, 0.88) ^#^	0.77 (0.57, 1.07)	0.60 (0.42, 0.85) ^#^	0.02
P value	-	0.01	0.12	0.01	-
Multivariable-adjusted OR (95%CI)	1.00 (reference)	0.65 (0.47, 0.90) ^#^	0.79 (0.57, 1.10)	0.64 (0.43, 0.94) ^#^	0.08
P value	-	0.01	0.16	0.02	-
Median vitamin E intake (mg/day)	15.97	22.80	30.71	45.93	-
MS (%)	16.2	17.1	15.5	19.0	-
Energy adjusted OR (95%CI)	1.00 (reference)	1.04 (0.74, 1.44)	0.89 (0.63, 1.26)	1.05 (0.70, 1.56)	0.93
P value	-	0.84	0.51	0.83	-
Multivariable-adjusted OR (95%CI)	1.00 (reference)	1.07 (0.77, 1.50)	0.98 (0.67, 1.41)	1.20 (0.77, 1.87)	0.46
P value	-	0.68	0.89	0.42	-
Median selenium intake (μg/day)	23.15	35.36	48.03	73.04	-
MS (%)	19.0	12.9	17.8	18.2	-
Energy adjusted OR (95%CI)	1.00 (reference)	0.60 (0.43, 0.85) ^#^	0.83 (0.59, 1.16)	0.75 (0.49, 1.14)	0.45
P value	-	0.00	0.28	0.18	-
Multivariable-adjusted OR (95%CI)	1.00 (reference)	0.60 (0.43, 0.86) ^#^	0.82 (0.58, 1.17)	0.72 (0.46, 1.14)	0.39
P value	-	0.01	0.27	0.16	-

MS: metabolic syndrome, OR: odds ratio, 95%CI: 95% confidential interval, multivariable adjusted OR: adjusting by age, sex, cigarette smoking, alcohol drinking, nutritional supplementary, activity level, dietary energy intake, fiber intake and protein intake.

^#^ P<0.05.

**Fig 1:**
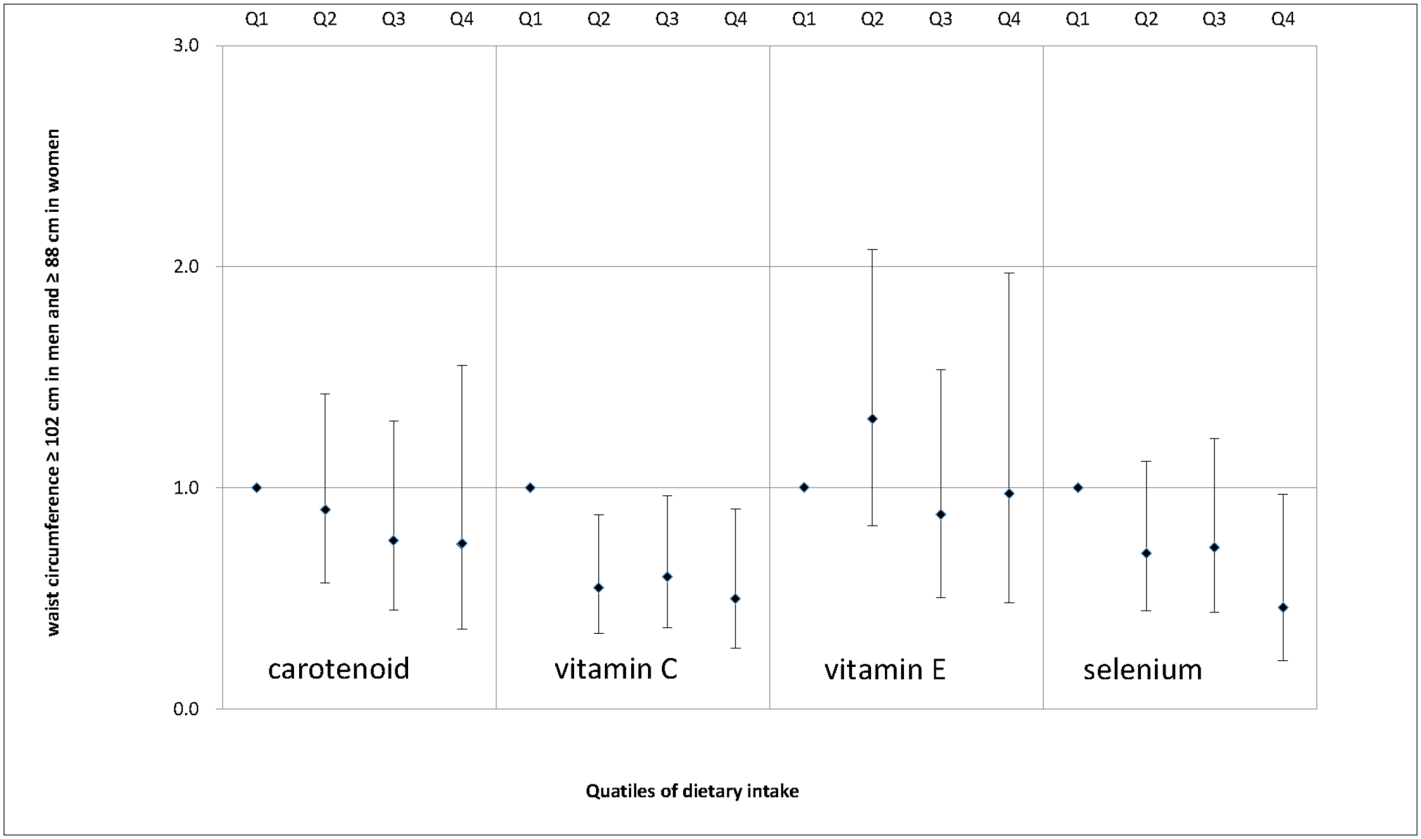
Adjusted OR and related 95% CI of dietary antioxidants intake (expressed as quartiles, Q2, Q3, Q4 vs. Q1) and enlarged waist circumference.

**Fig 2:**
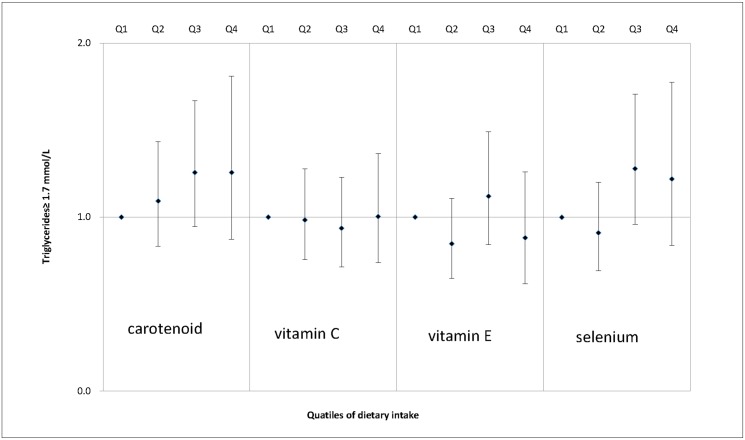
Adjusted OR and related 95% CI of dietary antioxidants intake (expressed as quartiles, Q2, Q3, Q4 vs. Q1) and elevated serum triglycerides.

**Fig 3:**
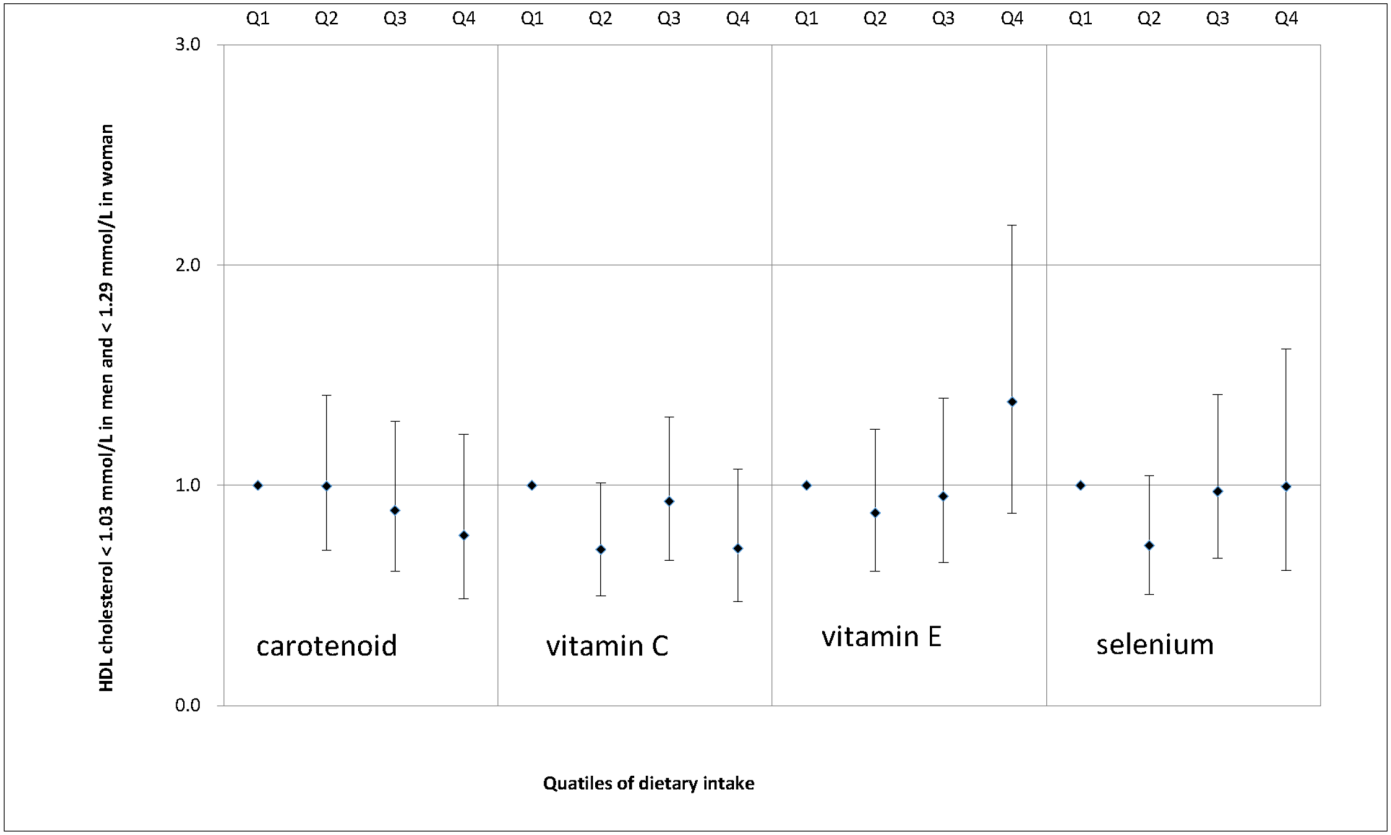
Adjusted OR and related 95% CI of dietary antioxidants intake (expressed as quartiles, Q2, Q3, Q4 vs. Q1) and decreased serum HDL cholesterol.

**Fig 4:**
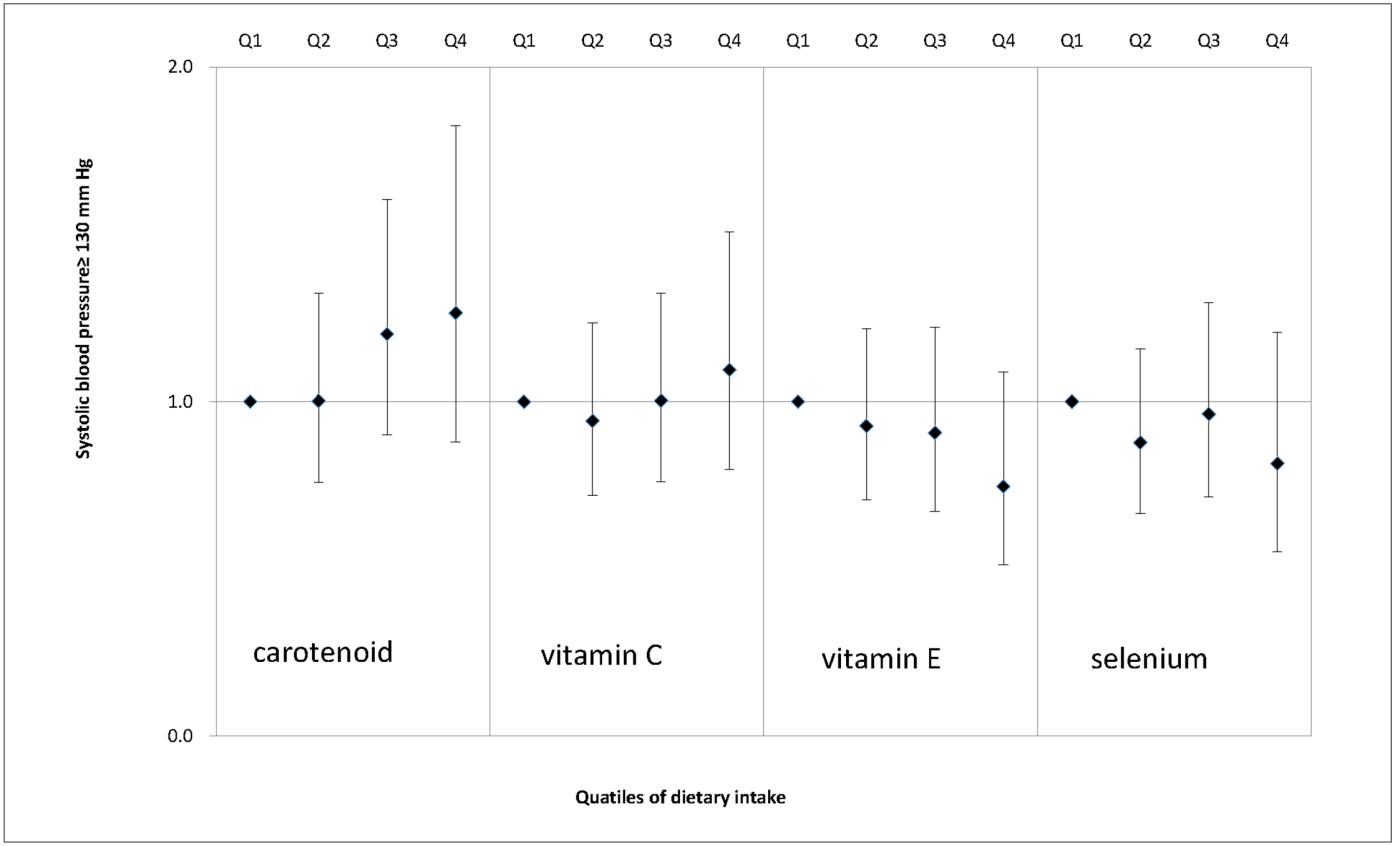
Adjusted OR and related 95% CI of dietary antioxidants intake (expressed as quartiles, Q2, Q3, Q4 vs. Q1) and elevated systolic blood pressure.

**Fig 5:**
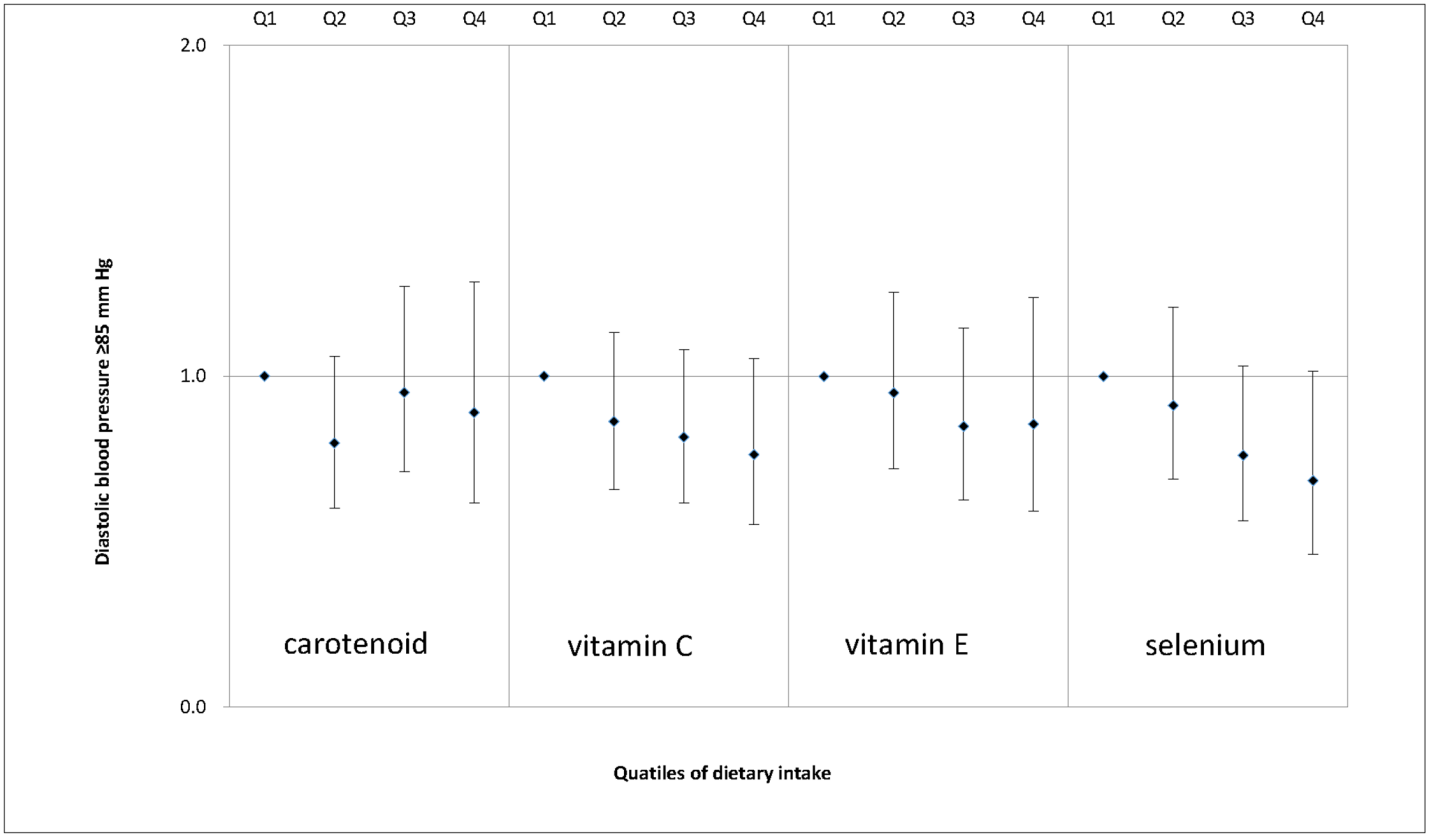
Adjusted OR and related 95% CI of dietary antioxidants intake (expressed as quartiles, Q2, Q3, Q4 vs. Q1) and elevated diastolic blood pressure.

**Fig 6:**
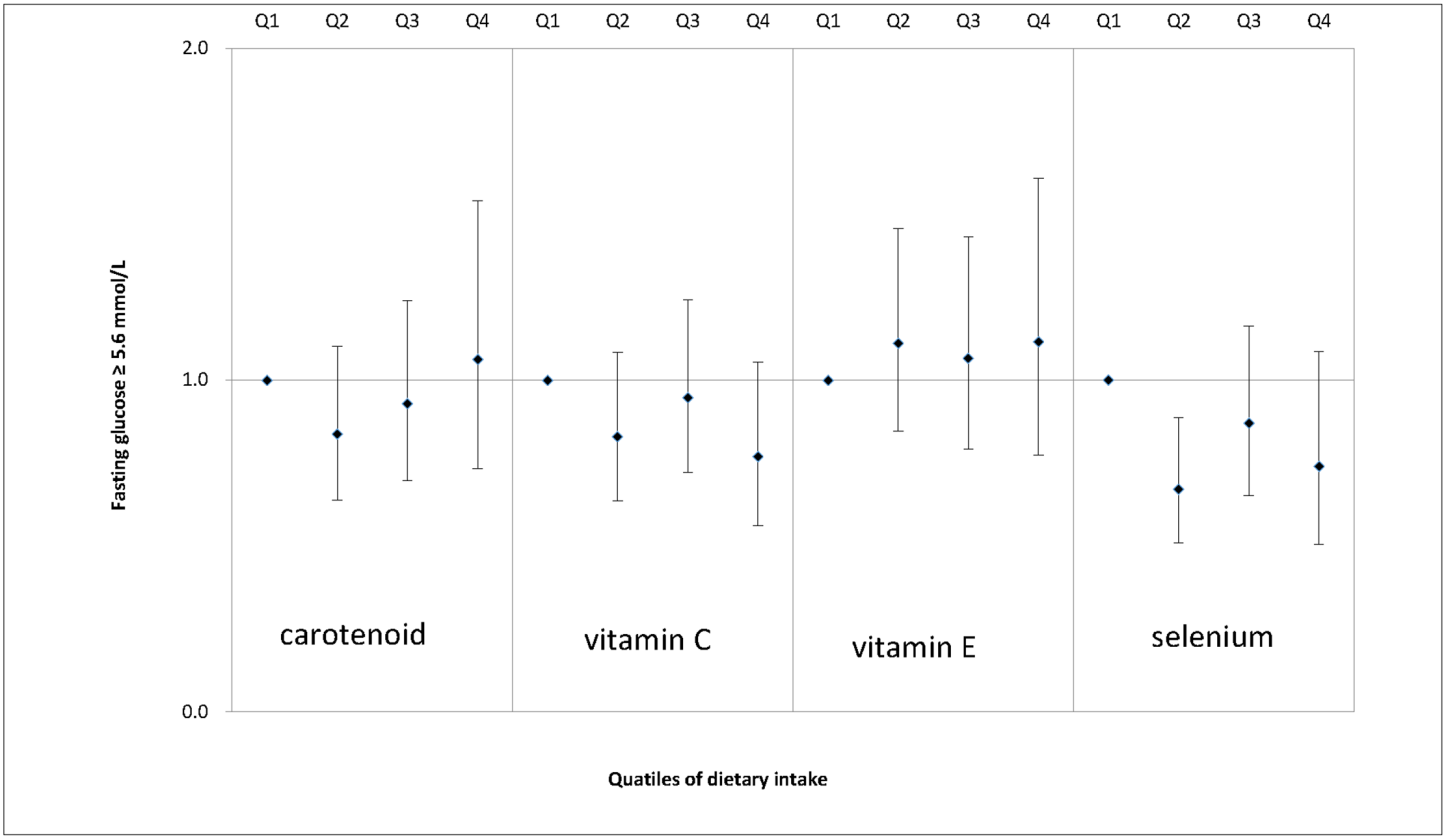
Adjusted OR and related 95% CI of dietary antioxidants intake (expressed as quartiles, Q2, Q3, Q4 vs. Q1) and elevated fasting glucose.

We also examined the associations between dietary antioxidant intake and MS features, including enlarged waist circumference (Fig 1), elevated serum triglycerides (Fig 2), decreased serum HDL cholesterol (Fig 3), elevated SBP (Fig 4), elevated DBP (Fig 5) and elevated fasting glucose (Fig 6). Both vitamin C and selenium intake showed a negative correlation with waist circumference (P values for trend: in both = 0.05). Selenium intake had a negative association with high DBP (P for trend = 0.04) and was also negatively correlated with hyperglycemia (OR = 0.67, 95%CI: 0.51 to 0.89, P = 0.01) in the second quartile compared with the reference quartile. Associations between dietary antioxidant and the other features of MS were absent in the multivariable logistic regression analysis.

## Discussion

A cross-sectional study with a large sample size (n = 2069) was performed to investigated the associations between dietary antioxidant intake (carotenoid, vitamin C, vitamin E and selenium) and MS. The prevalence of MS in the study population was 17.0%, which is similar to that previous reported in China (13.2% -15.8%) [3–6].

An increasing number of studies have examined the effects of inflammation and oxidative stress on several chronic diseases, including MS, diabetes, and cardiovascular disease. [7–10,19–23] Increased oxidative stress and impaired antioxidant defense were shown to have adverse effects on obesity, type 2 diabetes and atherosclerosis. [7,24,25] Subjects diagnosed with MS have a higher level of markers of oxidative stress and inflammation such as C3. [26,27] Additionally, elevated oxidative stress was positively associated with insulin resistance and compensatory hyperinsulinemia. [23,26]

Because oxidative stress plays such a crucial role in MS, there has been increased attention on the status of several antioxidants in subjects with MS. Puchau et al. [8] demonstrated that total dietary antioxidant capacity may be a potential early estimate of the risk of developing features of MS. The present study suggested that vitamin C intake had a significant negative association with MS, which is consistent with a previous study. [28] Lower intake of vitamin C may indicate an unhealthy diet habit, such as insufficient intake of vegetables and fruit, which may increase the risk of MS development. Dietary selenium intake was found to have a moderate association with MS, in our study. When selenium intake was compared with the reference, a negative association was observed, but only in the second quartile. Selenium has been suggested to be an important factor in cell defense against oxidative stress via the effect of antioxidant selenium-dependent enzymes. [29] However, the therapeutic range of selenium is relatively narrow, and some selenium compounds may generate toxic reactive oxygen species [30]. Previous studies show inconsistent conclusions related to the association between selenium and MS. Zulet et al. [28] concluded that selenium intake was negatively associated with SA (an inflammatory marker) and triacylglycerol levels. Another study explored the relationship between dietary selenium intake and risk of type 2 diabetes in northern Italy [31], and the investigators in that case concluded that increased dietary selenium intake was associated with an increased risk of type 2 diabetes. Additionally, Mutakin et al. [32] suggested that the association between selenium nutritional status and metabolic risk factors was limited to a particular group of obese men with or without MS.

A previous study concluded that serum carotenoid concentrations were inversely associated with MS status, but no significant difference of dietary β-carotene intake was found between MS patients and healthy participants [21], which is consistent with the current study. Ford et al. [14] concluded that subjects with MS had a significantly lower level of dietary vitamin A intake, but the total carotenoid intake was similar in the two compared groups; Zulet et al. also suggested that vitamin A intake was related to several anthropometrical and biochemical measurements that were linked to MS manifestations in healthy young adults, but they did not evaluate the association between pro-vitamin A intake and MS. [33] Our study also found no significant association between dietary vitamin E intake and MS, and this conclusion is consistent with several former studies [14,21]. Several other epidemiological studies did not find a significant association between dietary antioxidants intake and MS: Bian et al. [11] demonstrated that there was no negative correlation between the dietary antioxidant vitamins group and MS, Motamed et al. [13] and Li et al. [12] also found no significant association between dietary antioxidant intake and MS. Additionally, a few studies have suggested that antioxidant supplementation had no beneficial effects on MS [34,35].

Two similar studies in China had previously examined the relationship between dietary antioxidants intake and MS. [11,12] Compared with these two former studies, the present study exhibited several differences and strengths. First, the sample size in our study was much larger (2069 subjects), which allowed us to identify more significant associations between dietary antioxidants intake and MS. Second, we consider the results of the present study to be more reliable. Two models (energy adjusted and multivariable adjusted) were used and the resultant data were consistent. Additionally, our study examined the associations between dietary antioxidant intake (including dietary carotenoids, selenium, vitamin C, and vitamin E) and specific features of MS, which could provide a better understanding about their relationships.

There are, however, a few limitations existing in the present study. First, because the cross-sectional design was unable to explain any causal relationship, further prospective studies are needed to confirm our conclusions. Second, the biochemical status of serum, plasma, hair, or nail antioxidant was not measured in the target population in this study. Although, assessment of dietary antioxidant intake do not have the same accuracy as biochemical testing, dietary intake is important to human health. A review [36] demonstrated the condition of dietary selenium intake and selenium status in Europe and the Middle East. A total of 19 European/UK studies and 15 investigations in the Middle Eastreported selenium intake and selenium concentration in water and/or food. Eight of them assessed the dietary selenium intake using FFQ. A combined analysis of the relationships among dietary antioxidants, serum antioxidants and MS may provide a more comprehensive understanding of this topic.

## Conclusion

In conclusion, the prevalence of MS in the study population was 17.0%. According to the multi-variable adjusted analysis, subjects with low intake of vitamin C might be predisposed to development of MS, and dietary selenium intake had a moderate negative association with MS. However, dietary carotenoids and vitamin E intake were not associated with MS.
